# Surgical treatment of the severely damaged atlantoaxial joint with C1–C2 facet spacers: Three case reports

**DOI:** 10.1097/MD.0000000000015827

**Published:** 2019-05-31

**Authors:** Hiroyuki Tominaga, Anna MacDowall, Claes Olerud

**Affiliations:** a Department of Orthopaedic Surgery, Graduate School of Medical and Dental Sciences, Kagoshima University, Japan; b Department of Surgical Sciences, Uppsala University, Uppsala, Sweden.

**Keywords:** basilar invagination, C1–C2 facet spacer, coronal malalignment, occipital neuralgia, severely damaged atlantoaxial joint

## Abstract

**Rationale:**

Atlantoaxial subluxation (AAS), caused by congenital factors, inflammation such as rheumatoid arthritis, infection, neoplasia, or trauma, is rare and severely erodes and subluxates atlantoaxial (AA) joints. For these patients, surgical reduction, and stabilization are difficult. Surgery, including anterior transoral decompression and posterior fixation, anterior endonasal decompression and fixation, and posterior decompression with AA or occipitocervical fixation, is often the only treatment available. However, there have only been 2 reports of C1–C2 facet spacer use in treating AAS. Here, we report the case histories of 3 patients with severely damaged and subluxated AA joints and symptomatic basilar invagination (BI), malalignment, or C2 root compression.

**Patient concerns:**

The cases included 2 women with rheumatoid arthritis and 1 man with spondyloarthropathy secondary to ulcerative colitis.

**Diagnosis:**

Radiographic imaging revealed severely damaged and subluxated AA joints. Their symptoms included worsening pain in the neck or occiput with or without myelopathy and neuralgia.

**Interventions:**

After realignment with C1–C2 spacers and posterior C1–C2 screw fixation, the patient symptoms were resolved.

**Outcomes:**

Of note, 2 of the 3 patients were healed without complications. One patient who underwent secondary revision surgery because of rod breakage and obvious nonunion at C0–C2 was determined to be healed at 1-year follow-up after the revision surgery.

**Lessons:**

We confirmed that C1–C2 facet spacers both reduced BI and occipitocervical coronal malalignment as well as releasing C2 root compression. Therefore, surgical restoration and fixation should be a required treatment in this very rare group of patients.

## 1 Introduction

The etiology of atlantoaxial subluxation (AAS) may be congenital, inflammatory, infectious, neoplastic, or traumatic, although the cause is commonly multifactorial.^[1]^ Inflammatory lesions that most commonly cause AAS result from rheumatoid arthritis (RA). Cervical lesions are found in 42% of patients with RA,^[2]^ and atlantoaxial (AA) instability is present in 10% to 25% of RA patients.^[3,4]^ Bony erosion occurs early and progresses over time to involve destruction of the cruciate ligament of the atlas, and this segment loses stability, causing AAS to be evident 4 years after RA diagnosis.^[4]^ Further erosion and pannus formation in the odontoid process may cause the cranium to settle caudally and the dens to appear to enter the foramen magnum, which is termed basilar invagination (BI).^[5]^ BI causes compression of the spinal cord or brainstem,^[6]^ increasing the mortality rate eight-fold compared with RA patients without BI.^[7]^ The progression of cervical spine instability in RA correlates with Steinbrocker stage III or IV (Steinbrocker is a 4-staged index that describes the progression of RA),^[8]^ corticosteroid treatment, and previous joint surgery.^[9]^ Modern disease modifying anti-rheumatic drug (DMARD) therapy effectively achieves high remission rates and diminishes inflammatory activity, thus reducing the incidence of severe AAS.^[10]^ Nonetheless, a small number of patients still develop this condition where surgery is the only optional treatment to restore anatomy and reduce the compression of the spinal cord.

Different techniques to surgically treat AAS have been described, including anterior transoral decompression and posterior fixation,^[11]^ anterior endonasal decompression and fixation,^[12]^ and posterior decompression with AA,^[13]^ or occipitocervical fixation.^[14]^ In rare cases of severe destruction of the AA joint with or without BI, it is difficult to reduce the subluxation. Restoration of the anatomy and alignment with C1–C2 facet spacers before stabilization with posterior screw fixation has been described. However, information about the method's feasibility is sparse and few cases with slight derangement of the AA joint have been reported.^[15]^ The aim of this study was to highlight the surgical technique using C1–C2 facet spacers for the reduction and alignment of AA joints with severe destruction and subluxation by presenting a case series of 3 treated patients.

## 2 Methods

### 2.1 Symptoms and signs

Symptoms of myelopathy may include neck pain and stiffness, paresthesia, and long tract signs in the lower extremities, such as gait/balance difficulties, hyperreflexia, muscle weakness, and Lhermitte's sign, as well as Hoffman's sign in the upper extremities. C2 root compression may manifest with symptoms such as occipital neuralgia, which is an intense pain described as a sharp, jabbing, electric shock in the back of the head and neck.^[16]^

Computed tomography (CT) may reveal lateral subluxation of the AA joint, odontoid fracture, destruction of the AA joint, vertical subluxation of the axis, BI, and subaxial subluxation.^[9]^ The vertical atlantoaxial index (VAAI) measures the vertical relationship of the atlas and axis, thus quantifying the degree of decompression achieved when a reduction of the subluxation of the AA joints is performed with facet spacers as previously described: “A horizontal line is drawn through the lower endplate of axis. A second line is drawn parallel to this and tangential to the lower border of the anterior arch of atlas. Furthermore, a third line is drawn parallel to these lines and tangential to the superior margin of dens. The shortest distance between the first 2 lines (x) is divided by the shortest distance between the first and third lines (y).” The normal index value is 0.8 (range, 0.76 to 0.85).^[17]^

Preoperative magnetic resonance imaging (MRI) may reveal spinal cord compression by the atlas.

### 2.2 Surgical technique

Cervical traction with a force of 4 to 5 kg prior to surgery prepares the AA joint and facilitates reduction and realignment. The patient is placed in a prone position with the head end of the table slightly elevated. Lowering the peak end-expiratory pressure may decrease venous bleeding during exposure of the AA joint. A standard posterior approach to the C1–C2 is performed. The C1–C2 facet joint is distracted with a Cobb elevator. The status of the subluxation and of BI is evaluated by intraoperative radiography,^[5]^ or O-arm (Medtronic) images. The spacer is inserted in the collapsed joint, with care to preserve the C2 root. The spacer we used is the Corridor anterior cervical cage (Globus Medical Inc., Audubon, PA) because of its sufficiently small footprint.

Because this device is not designed to be a facet spacer but rather a cervical cage for anterior fusion, we used the smallest size available. The Corridor anterior cervical cage is CE marked (CE=Conformité Européenne, a certification mark that indicates conformity with the health, safety, and environmental protection standards for products sold within the European Economic Area, but is not approved by the US Food and Drug Administration.

After reduction and realignment with the facet spacer, we performed C1–C2 fixation with a C1 claw,^[18]^ or a C1 lateral mass screw,^[19,20]^ without a C2 root transection,^[21]^ and a C2 Goel–Harms screw. In irreducible cases, we performed occipito-cervico-thoracic fixation. A corticocancellous bone graft was harvested from the iliac crest.

Possible intraoperative complications with the procedure include damage to the C2 root, injury to the vertebral artery and misplaced screws. Possible postoperative complications include infection, delayed fusion or pseudarthrosis.

### 2.3 Follow-up

Routine follow-up was performed 3 to 4 and 6 to 8 months after surgery, with the variation depending on available appointments combined with patient preference. When required, the patient was followed-up to 1-year to confirm bony fusion and symptom release. There was also a postal follow-up at 1-year after surgery with patient-reported outcome measurements (PROM) including the European Myelopathy Score (EMS) and the visual analog scale (VAS) for neck and arm pain and global assessment.

Myelopathy was measured using the EMS, a patient-reported function score that ranges from 5 to 18 points, with lower scores indicating greater disability. The questions concern 5 items (gait function, bladder and bowel function, hand function, proprioception and coordination, and paresthesia/pain): 17 and 18 points are considered normal; 13 to 16 points are myelopathy grade 1; 9 to 12 are myelopathy grade 2 and; 5 to 8 are myelopathy grade 3.^[22]^

The VAS for neck and arm pain ranges from zero to 10, with higher scores indicating more severe pain.^[23]^ The MCID is 2.5 for VAS of the neck and for VAS of the arm.^[24]^

Global assessment in the follow-up questionnaires was measured using 3 grades: symptoms improved after surgery, no change in symptoms after surgery or symptoms deteriorated after surgery.

## 3 Case reports

### 3.1 Case 1

**Figure 1:**
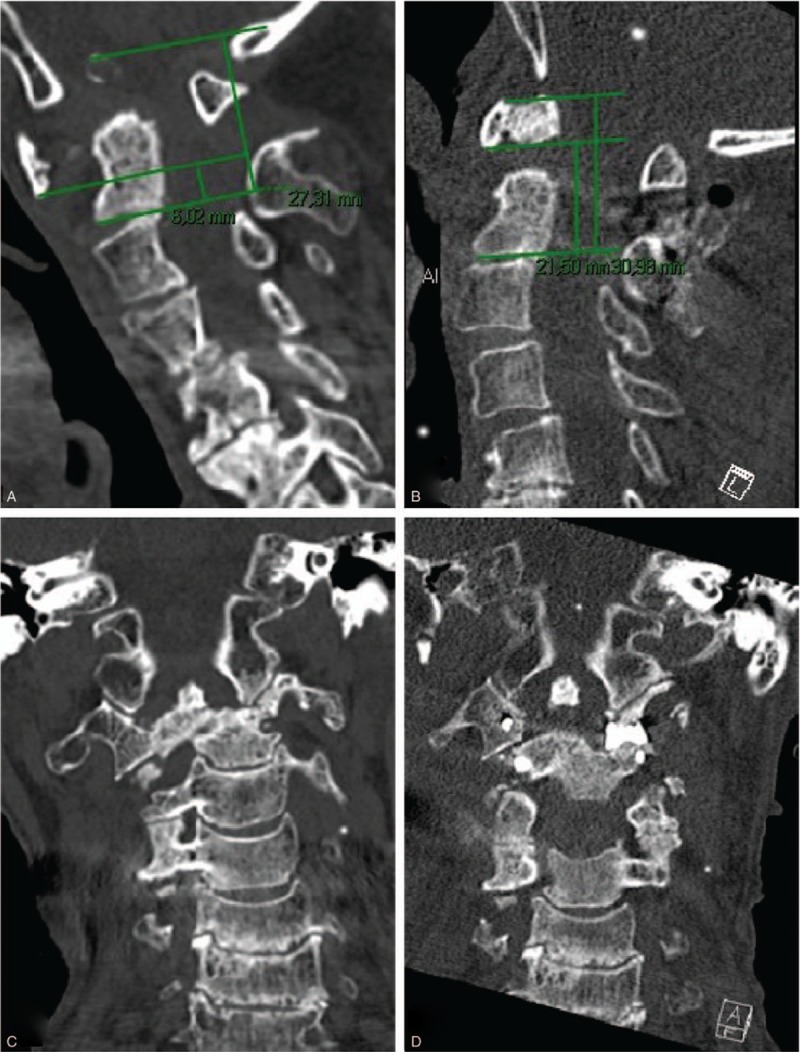
Images from case 1. (A) Preoperative sagittal CT view. The lines required to measure VAAI are shown. VAAI = 0.22. (B) Preoperative coronal CT view. The C1–C2 facet joint on the left side is severely damaged, with the axis shifting to the left. The axis is shifted posteriorly with basilar invagination. (C) Postoperative sagittal CT view. The lines required to measure VAAI are shown. VAAI = 0.69. (D) Postoperative coronal CT view. The C1–C2 facet spacer is inserted on the left side, improving coronal alignment. CT = computed tomography, VAAI = vertical atlantoaxial index.

Patient 1 was an 85-year-old woman, height 166 cm, weight 39 kg, and who smoked 10 cigarettes/day. She was retired but had worked fulltime at a desk job until the age of 65 years. She had RA with severe destruction and deformation of the joints in her hands, fingers, wrists, shoulders, hips knees, ankles, and feet. Her hands were especially affected and all the fingers on both hands were subluxated, partially preventing her from using a pen, fork, or dress. She had suffered from neck pain for more than 2 years. Over a couple of weeks, she had rapidly lost the ability to walk, was EMS 9 (grade 2), and her neck pain had increased to unbearable (VAS 10), although she had no arm pain. At physical examination, she presented with cervical myelopathy (Ranawat 3b^[25]^). Her legs were weak, and although she could move them when lying in bed she was unable to stand or walk. Hoffman's sign was positive, and her lower extremities showed hyperreflexia. Preoperative CT revealed vertical instability, VAAI 0.22 (Fig. 1A), with severe destruction and lateral subluxation of the AA joint (Fig. 1B) and odontoid fracture. Preoperative MRI revealed spinal cord compression by the atlas. Cervical traction with 4 kg for 2 days improved the patient's neurological symptoms and gross alignment. We performed C1–C2 fusion with a spacer in the right AA joint (Fig. 1D), a C1 claw (right side), a C1 lateral mass screw (left side), and bilateral C2 pedicle screws. The patient's myelopathy improved without C1 decompression (postoperative VAAI 0.69, Fig. 1C). At 4 months follow-up the patient could walk 100 m with a walker and at 7 months the patient's neck pain was reduced to VAS 2. Her EMS had improved to 13 (grade 1) and the global assessment was “symptoms improved after surgery”.

### 3.2 Case 2

**Figure 2:**
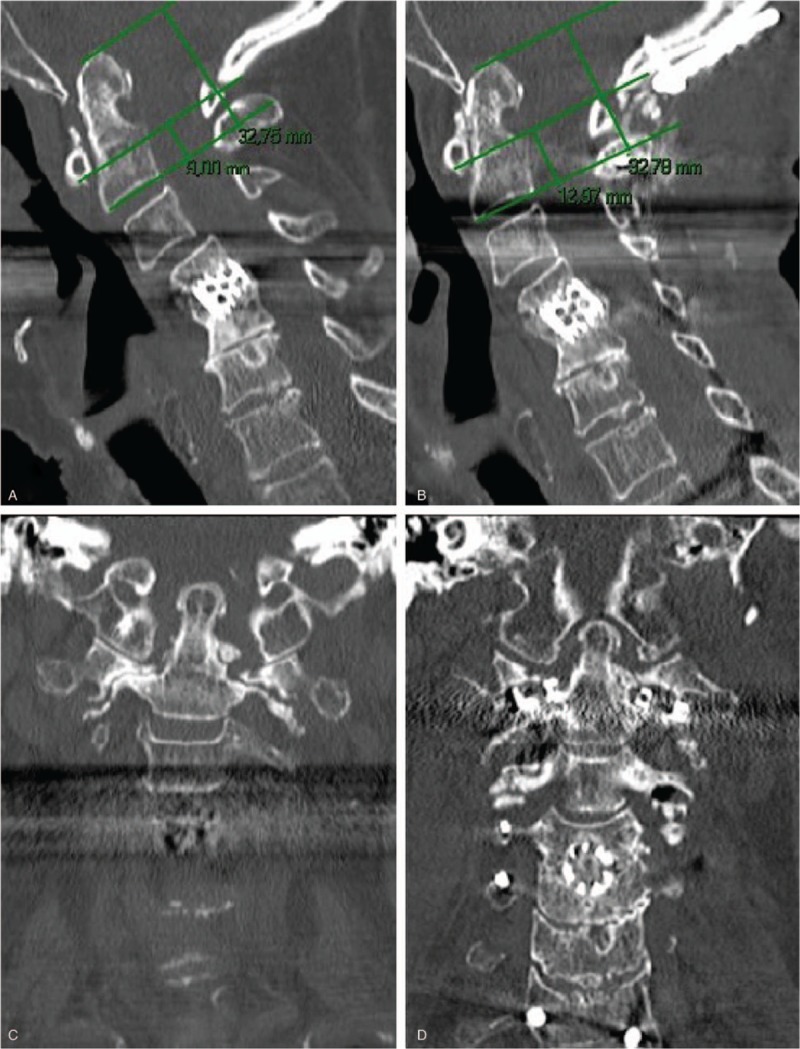
Images from case 2. (A) Preoperative sagittal CT image. The lines required to measure VAAI are shown. VAAI = 0.27. (B) Preoperative coronal CT image. (C) Postoperative sagittal CT image. The lines required to measure VAAI are shown. VAAI = 0.402. (D) Postoperative coronal CT image. The C1–C2 facet spacers are inserted bilaterally. CT = computed tomography, VAAI = vertical atlantoaxial index.

Patient 2 was a 75-year-old woman, height 157 cm, weight 56 kg, a nonsmoker who was retired and diagnosed with RA at 40 years of age. She had previous surgery at C4–C5 related to disc degenerative changes with radiculopathy. She had suffered from neck pain for more than 2 years but the pain had increased over the past few months, and she had started to suffer from neck pain on rotation and occipital neuralgia (missing values for VAS). She had no arm pain but experienced a deterioration of fine motor skills. She could walk 100 to 500 m and had an EMS of 11 (grade 2). At physical examination, she had no symptoms of myelopathy and her restricted walking distance and disability of fine motor skills were concluded to be caused by RA related destruction and deformation of the joints. Preoperative images revealed AA subluxation and BI (VAAI 0.27, Fig. 2A, B).

We performed C0–T2 fusion, lifting the AA joints bilaterally with facet spacers, increasing the VAAI to 0.40 (Fig. 2C, D). At 3 months of follow-up, the patient's symptoms had improved and at 7-months of follow-up, she had no headache, neck pain and no symptoms of pathologic neurology.

Eleven months after surgery the patient had a sudden onset of instability and neck pain. A CT scan revealed breakage of the right rod and obvious nonunion at C0–C2. A reoperation was performed with rod exchange and bone graft from the iliac crest together with bone morphogenetic protein (BMP). At 3-months of follow-up, the symptoms of instability and neck pain had resolved. At 1-year of follow-up after revision surgery, her EMS was 10 points (grade 2) and the global assessment was “symptoms improved after surgery”. Her walking distance was now less than 100 m and her fine motor skills had deteriorated because of RA progression-related changes in her hands and feet.

### 3.3 Case 3

**Figure 3:**
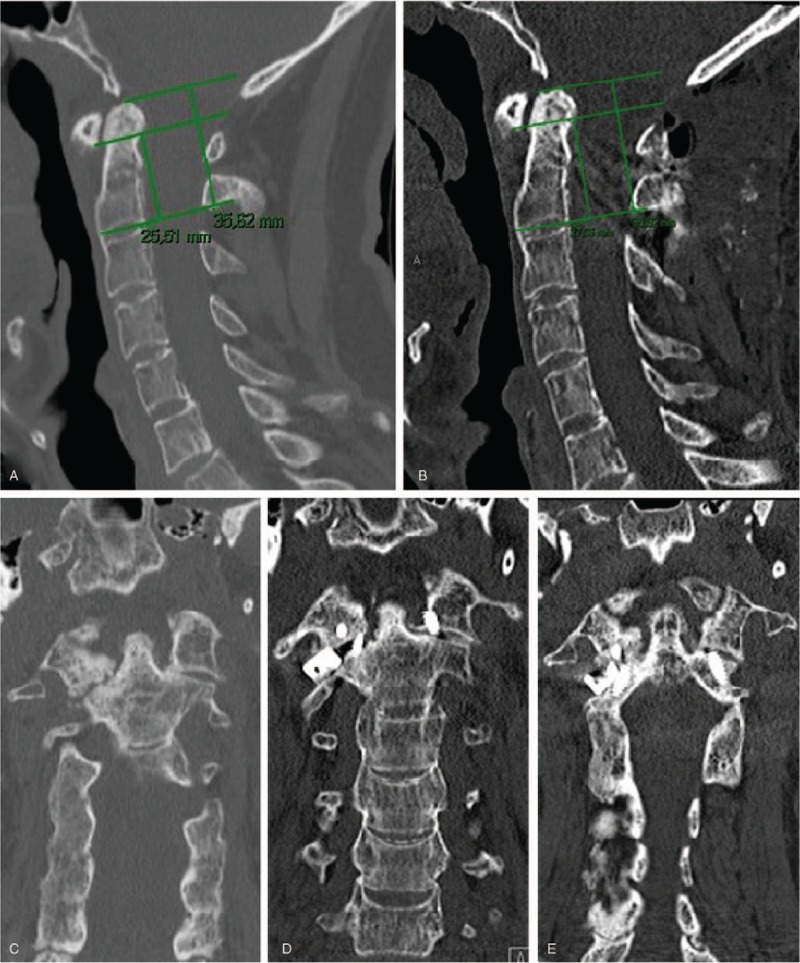
Images from case 3. (A) Preoperative sagittal CT image. The lines required to measure VAAI are shown. VAAI = 0.72. (B) Preoperative coronal CT image. The C1–C2 facet joint is severely damaged on the right side. (C) Postoperative sagittal CT image. The lines required to measure VAAI are shown. VAAI = 0.76. (D) Postoperative coronal CT image. The C1–C2 facet spacer is inserted on the right side and coronal alignment was improved. (E) Coronal CT image at 4 months follow-up. Callus formation and bony fusion in the right C1–C2 facet are present. CT = computed tomography, VAAI = vertical atlantoaxial index.

Patient 3 was a 43-year-old man, height 173 cm, weight 80 kg, who was a nonsmoker with spondyloarthropathy secondary to ulcerative colitis and sclerosing cholangitis presenting with very severe neck pain that worsened on rotation and head tilt that required opioid medication. He was working full-time with a desk job and was not on sick leave. The duration of neck pain was 1–2 years but had recently increased over the past few months (VAS 6). He had no arm pain and his fine motor skills were good/normal. He could walk more than 1 km and his EMS was 18 points (normal). At physical examination, there were no signs of neuropathy or myelopathy. Images revealed osteoarthritis and destruction of the right AA joint (Fig. 3A, B). We performed C1–C2 fusion to relieve his neck pain and used a joint spacer to balance the head. Traction was applied during surgery but no preoperative skull traction was performed. At 4-months follow-up, the patient had no ongoing neck pain and bony fusion was confirmed with CT. At 1-year of follow-up, the patient was working full-time at the same job, and walking more than 1 km with an EMS of 17 points (normal), VAS for neck pain of 0, with fine motor skills that were good/normal and a global assessment of “symptoms improved after surgery”.

## 4 Discussion

This is the first case series describing the reduction and realignment of AA joints with severe destruction and subluxation using C1–C2 facet spacers since the initial description of the technique by Goel et al. The index level was stabilized with posterior screw fixation connected with rods, which was described as a safe and sustainable method for up to 14 years of follow-up.^[26]^ Mortality rates among patients with RA and BI have been reported to be 20% at 5 years and 33% at 10 years after surgery,^[27]^ and without surgical treatment, the estimated 1-year mortality rate was 50%.^[28]^ Therefore, surgical restoration and fixation is not optional but is lifesaving in this rare group of patients. The best surgical technique to accomplish realignment, as well as the shortest possible instrumentation, will enhance the patient's quality of life.

When AA joints are severely damaged asymmetrically (grade 3),^[29]^ as in cases 1 and 3, the cervical spine starts to collapse on 1 side, creating a coronal deformity that may be challenging to restore. Bilateral destruction of AA joints may cause the cranium to settle caudally, as in BI, which compresses the spinal cord (cases 1 and 2) and/or the C2 roots (cases 2 and 3). Various techniques to surgically treat severely damaged AA joints transorally or posteriorly with decompression and fusion have been described,^[12,14]^ without addressing the main problem of the damaged and collapsed AA joints. The C1–C2 facet reduction technique with insertion of spacers restores alignment by lifting the AA joints into place and consequently stabilizing the cranium.^[5,30]^ This allows a shorter fusion involving only the index level without the need to include the remaining cervical levels. With reduction and restored alignment, both myelopathy (case 1) and occipital neuralgia (cases 2 and 3) were improved, even though no anterior or posterior decompression was performed. We prefer C2 root preservation instead of transection when distracting the C1–C2 facet joints and inserting the spacers, as previously described,^[21]^ allowing C2 root compression to be relieved when the C2 neural foramina is lifted. Although the use of posterior screw and rod fixation prevents the spacer from severe dislocation and loosening, the spacer tends to slide posteriorly and laterally and needs to be positioned more anterior (case 1) and medial (case 3) than anticipated. In case 3, the spacer showed lateral displacement on postoperative CT but the spacer still lifted the joint several mm (Fig. 3D) and there was a successful bony fusion 4 months after surgery (Fig. 3E). It is challenging to insert the spacer perfectly because of rigid deformity and severe joint destruction and the facet space needs to be spread for the inclination to be reduced. Even a spacer that is not perfectly centered can achieve a reduction of the subluxation and coronal realignment when combined with stabile fixation and osseous healing that preserves the realignment from future continuous joint destruction and subluxation.

This study had several limitations. First, there was no control group because it was a case series. Second, the follow-up periods were short and therefore, longer observation times are required. Third, this series included only 3 patients. Although the inclusion of more patients would have been preferable, patients with severely damaged AA joints caused by RA are rare.

Reports of the use of C1–C2 spacers in RA patients are rare and the only case series previously described was the inventor of the technique.^[5,15]^ In this report, we have provided additional information about the usefulness and versatility of this technique and have included the CT findings of 3 patients. Because this was a case series with 3 patients and not a comparative study, none of the patients suffered from intraoperative or severe complications. The technique of reducing BI and occipital-cervical-coronal malalignment with C1–C2 facet spacers appear useful, but further studies with additional cases and longer follow-up are necessary to establish its safety and effectiveness.

## Author contributions

**Conceptualization:** Claes Olerud.

**Supervision:** Claes Olerud.

**Writing – original draft:** Hiroyuki Tominaga.

**Writing – review & editing:** Anna MacDowall.
